# Antimicrobial Resistance in Endemic Enteric Infections in Kenya and the Region, and Efforts Toward Addressing the Challenges

**DOI:** 10.1093/infdis/jiab457

**Published:** 2021-09-22

**Authors:** Samuel Kariuki, Celestine Wairimu, Cecilia Mbae

**Affiliations:** Centre for Microbiology Research, Kenya Medical Research Institute, Nairobi, Kenya

**Keywords:** Sub-Saharan Africa, Kenya, enteric infections, Multidrug-resistant, Genomics

## Abstract

Resistance to commonly available antimicrobials is a major threat to the fight against endemic bacterial diseases in sub-Saharan Africa, with a majority of the population unable to afford alternative effective antimicrobial options for management of these diseases. Diseases such as typhoid, cholera, and invasive nontyphoidal *Salmonella* are among the key enteric infections endemic in most parts of sub-Saharan Africa, especially in displaced populations and among the urban populations living in overcrowded informal settlements. Here, we explore the prevalence and the genomic epidemiology of these infections and the growing problem of multidrug resistance, including emerging resistance to the last line of treatment for these infections. Prevalence rates to commonly available antimicrobials, including ampicillin, chloramphenicol, cotrimoxazole, and tetracycline, now range between 65% and 80%, while 15%–20% of recently studied isolates show reduced susceptibility to fluoroquinolones and emerging resistance to extended-spectrum β-lactams mediated by the *CTX-M-15* gene carried on a highly mobile genetic element. The high prevalence of multidrug-resistant isolates including resistance to reserve antibiotics, calls for enhanced control and management options. It will be important for governments in the region to enhance the implementation of national action plans, as guided by the global action plan championed by the World Health Organization, to combat the threat of antimicrobial resistance. However, to yield meaningful results, these efforts will require a strong commitment and enhancement at all levels of healthcare in order. In addition, the use of World Health Organization–approved vaccines in the short to medium term and improvement of water and sanitation in the long term will reduce the burden of disease and antimicrobial resistance in the region.

Infectious diseases are among the 10 leading causes of global disease burden [1]. In Africa, they account for at least 69% of deaths on the continent, with enteric infections causing a disproportionally high morbidity and mortality rates in the sub-Saharan Africa (SSA) region [2, 3]. As the population in SSA grows and more people seek jobs in the urban areas, the challenges of overcrowding in informal settlements with poor water, sanitation, and hygiene infrastructure will continue to pose health challenges for the communities. In addition, conflicts have also led to displacement of huge populations that also end up in poor settlements, and these serve as epicenters of enteric infections such as cholera, *Salmonella,* and *Escherichia coli* diarrhea. Here, we describe the epidemiology and genomics of 3 key multidrug-resistant (MDR) enteric infections that are endemic to the region and the emergence of resistance to extended-spectrum β-lactams (ESBLs) and fluoroquinolones, which are among the last-resort antibiotics for many infections in the region. All the studies described here were approved by the Scientific and Ethics Review Unit (SERU) of the Kenya Medical Research Institute (SERU no. 2076). All parents and/or guardians of participating children were informed of the study objectives and voluntary written consent was sought and obtained before their enrolment.

## RAPID SPREAD OF MDR *S*. TYPHI HAPLOTYPE 58 IN SSA

Globally, typhoid fever caused by *Salmonella enterica* serovar Typhi is estimated to cause approximately 14.3 million illnesses and 135 900 deaths annually [4], 11.9 million of these cases, resulting in 129 000 (75 000–208 000) deaths, occur in low- and middle-income countries [5]. In SSA, the incidence of typhoid is now estimated at an annual average of 318/100 000 person-years of observation [6]. Previously, our group reported typhoid incidence at 263/100 000 person-years of observation (95% confidence interval, 199–347) in all age groups in Kenya [7].

In SSA, typhoid fever remains endemic partly because the supply of clean drinking water and sanitation have not kept pace with the rapid population growth in large cities, with most cases occurring in poor informal settlements and among displaced populations [8, 9]. The GenoTyphi scheme classifies *S*. Typhi genotypes into 4 primary clusters, which are further divided into 16 clades and 49 subclades based on single-nucleotide polymorphisms. Primary clades are designated 1.1 and 1.2, while subclades are designated 1.1.1 and 1.1.2. [10]. *S*. Typhi haplotype 58 (H58) (defined as genotype 4.3.1) is a globally disseminated subclade associated with MDR (defined as resistance to chloramphenicol, ampicillin, and cotrimoxazole), conferred by horizontally acquired genes, and increasing frequency of reduced susceptibility to fluoroquinolones, conferred by mutations in core genes *gyrA* and *parC* [11, 12]. *S*. Typhi H58 are rapidly displacing other lineages in many endemic areas of typhoid, initially in Southeast Asia, but now have spread globally to other endemic sites in SSA [10, 13].

**Figure 1.**
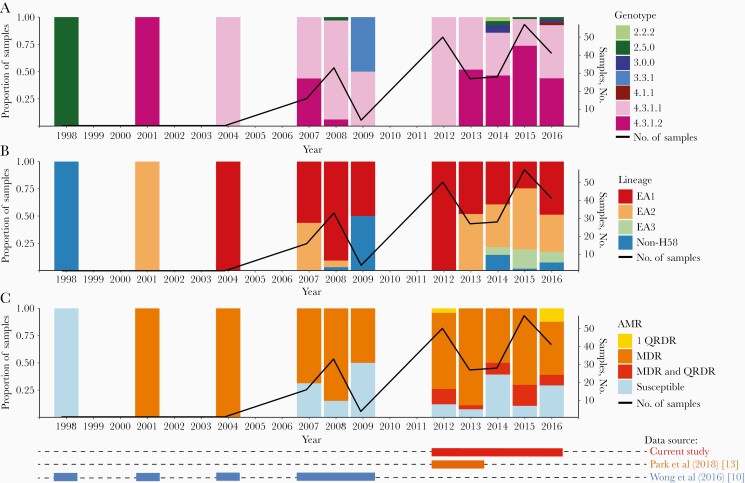
*Salmonella* Typhi trends in Kenya from 1995 to 2016 in the context of genotypes, lineages, and resistance to commonly available drug and fluoroquinolones. (These data have been compared with those from previous studies [10, 13].) Abbreviations: AMR, antimicrobial resistance MDR, multidrug resistant; QRDR, quinolone resistance determining region.

A critical feature of *S*. Typhi H58 in the SSA region is MDR to the commonly available drugs, which is a challenge for management of outbreaks in endemic areas. Current data from Kenya shows that only 18% of these isolates are fully susceptible to commonly available antimicrobials. The majority (60.5%) are MDR *S*. Typhi also resistant to all commonly available drugs and show reduced susceptibility to ciprofloxacin [7]. These strains belong to 2 main lineages, lineage I (genotype 4.3.1.1) (62.1% of H58) and II (genotype 4.3.1.2) (37.9%). These lineages are further subdivided into several sublineages, dependent on periods of introduction and subsequent mutations (Figure 1). The proportion of H58 has increased rapidly from 2004, and with it the proportion of MDR isolates with reduced susceptibility to fluoroquinolones.

## EPIDEMIOLOGY AND GENOMICS OF CHOLERA IN THE REGION

Globally, toxigenic *Vibrio cholerae* causes 1.4–4.3 million illnesses, resulting in approximately 21 000–143 000 deaths per year. Most of the cholera cases reported globally are due to the seventh cholera pandemic, driven mainly by the 01 El Tor biotype, which has persisted in most low- and middle-income countries, but SSA continues to bear the greatest burden of frequent outbreaks [14, 15].

**Figure 2.**
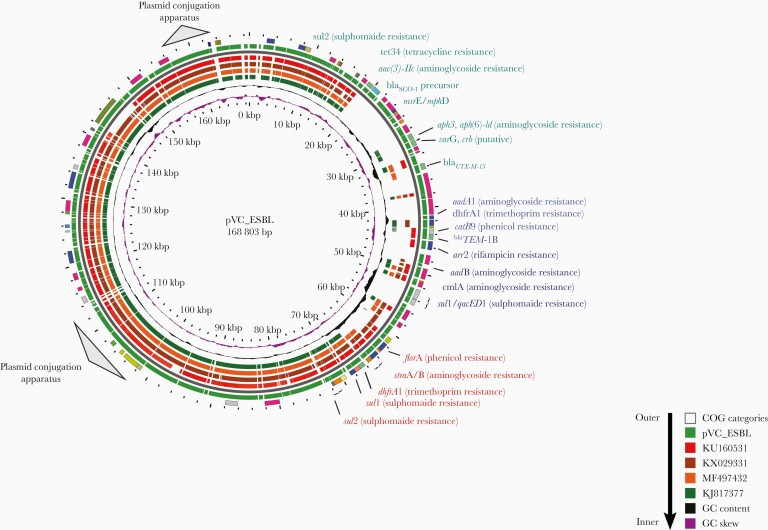
The plasmid_Vibrio Cholerae (pVC)_extended-spectrum β-lactam (ESBL) plasmid bearing various antimicrobial resistance genes. Different bar color codes show the comparison between new plasmid pVC_ESBL genes and previous plasmids from a global collection. Abbreviations: bp, base pair; COG, Clusters of Orthologous Genes; GC, guanine-cytosine; kbp, kilobase pair.

Cholera epidemics are partially driven by frequent outbreaks in refugee camps, where overcrowding is common, and in urban slums, where basic sanitation is lacking and clean drinking water is frequently in short supply. Although antibiotics are not always recommended for management of cholera, there has been an upsurge of MDR *V. cholerae* strains in Kenya and the region in the last 10 years [16, 17]. For instance, outbreaks have been reported in Uganda [18], Mozambique [19], Ghana [20], and South Africa [21], with strains being MDR to commonly used antimicrobials and also resistant to third-generation cephalosporins and fluoroquinolones. In Kenya, we recently detected and characterised MDR *V. cholerae* with genes encoding ESBL enzymes on a highly mobile genetic element (Figure 2).

These ESBL-producing isolates were resistant to chloramphenicol, streptomycin, sulfamethoxazole, trimethoprim, tetracycline, rifampicin, and erythromycin but were susceptible to doxycycline and ciprofloxacin. All isolates belonged to the sequence type (ST) 69 clone complex associated with the seventh pandemic O1 El Tor lineage. The ESBL-producing isolates carried an IncA/C conjugative plasmid, plasmid_Vibrio Cholerae (pVC)_ESBL, which contains a class 1 integron and an SXT R391–integrating conjugative element [7]. pVC_ESBL also carried an ISE*cp*-linked ESBL gene, *bla*_CTX-M-15_, which confers resistance to third-generation cephalosporins. The acquisition of the large mobile genetic element with the repertoire of resistance genes may be the result of interaction and transmission across bacterial genera and species in the human gut environment [22, 23].

The pVC_ESBL plasmid has 3 distinct resistance regions. The first of these contains the SXT R391 carrying the *floR*-*dhfr*A1-*strA*-*strB*-*sul*2 genes. The second contains a class 1 integron carrying *aadB*-*arr2*-*bla*_TEM1B_-*cmlA*-*bla*_OXA-10_-*arr*-2-*aadA1* cassettes and a 3’-conserved sequence element containing *sul1* and the truncated *qacEΔ*1 gene. The third region carries resistance genes inserted into the plasmid backbone, encoding resistance to third-generation cephalosporins (*bla*_CTX-M-15_, *aac(3)-IIc*) which confers resistance to streptomycin, kanamycin, and tobramycin; and also a putative gene for tunicamycin resistance.

These MDR strains with resistance genes carried on highly mobile genetic elements pose a major public health challenge in the management of these and other enteric infections in the region. It therefore calls for renewed efforts for control and management of cholera, including the introduction of routine vaccination in endemic settings, using World Health Organization (WHO)–approved oral cholera vaccines.

## MDR INVASIVE NONTYPHOIDAL *Salmonella* INFECTIONS IN CHILDREN

Globally, nontyphoidal *Salmonella* disease caused nearly 4 847 000 disability-adjusted life-years lost (70 disability-adjusted life-years/100 000 population) and 81 300 deaths (1.2 deaths/100 000 population) in 2010 [24]. In SSA, invasive nontyphoidal *Salmonella* (iNTS) disease is endemic and causes severe life-threatening illness, especially among vulnerable children <5 years of age (average incidence, 227 [range, 152–341] cases per 100 000 population). The SSA region accounts for the largest number of cases (1.9 [range, 1.3–2.9]) million cases) [25] with a case fatality rate between 20% and 25% [26–29]. Among iNTS cases, it is estimated that 63.7% occurred in children <5 years of age globally, and 68.3% in children <5 years of age in SSA [25]. The increasing rates of antimicrobial resistance (AMR) in iNTS is of great global concern, and the situation is even more acute in SSA, where empiric oral options for effective treatment of life-threatening invasive disease are being rapidly eroded.

MDR iNTS has been reported in Kenya and Malawi [30–32] and elsewhere in the SSA [33, 34] posing a major challenge to treatment and management options. Where new effective antimicrobials are lacking, developments in vaccines offer hope for reducing the burden of iNTS in endemic settings in SSA. Within the last decade, MDR *S.* Typhimurium of a novel ST, ST313, has been reported from several countries in SSA, including Kenya [30], Malawi [35], the Democratic Republic of the Congo [36], Nigeria [37], Ghana [38], and Mozambique [39], where iNTS is endemic and produces septicemia in the absence of gastroenteritis.

Whole-genome sequencing of strains circulating in the region has also shed light on the most prevalent genotypes for *S.* Typhimurium and *S.* Enteritidis in SSA. In several SSA countries, *S.* Typhimurium ST313 [40–43] and *S*. Enteritidis ST11 [32, 33, 43] are most commonly associated with invasive disease. Several countries have also reported on the emergence of MDR in these iNTS strains, some with resistance to ESBLs (eg, ceftriaxone), which are the last-line treatment options for severe invasive disease in the region. In Kenya, we observed a high incidence of MDR *S.* Typhimurium ST313, the genotype most commonly associated with increasing resistance to ceftriaxone [30, 43]. In addition, MDR *S.* Enteriditis ST11 strains have also has emerged in Kenya and continue to flourish at the expense of strains that are more susceptible [43]. The strain genotypes from case patients and did not differ significantly those from controls. In addition, the MDR genotype was equally distributed between case patients and controls, clearly indicating the importance of carriage in the maintenance and dissemination of MDR lineages.

## DIAGNOSTIC CHALLENGES LEADING TO MISUSE AND ABUSE OF ANTIMICROBIALS

Owing to the limited laboratory capacity in most healthcare facilities in the region, most infections are treated empirically [44]. This indirectly contributes to the emergence of AMR and poor patient treatment outcomes. In poor urban populations living in informal settlements where infectious diseases are endemic and access to healthcare is not optimal, people resort to self-medication, which this is deemed to be cheaper than seeking treatment at healthcare facilities. In addition, the availability of over-the-counter antimicrobials at numerous retail outlets in most of the densely populated informal settlements contributes to misuse and abuse of antimicrobials, again exacerbating the growing AMR pandemic in the region [45, 46]. The availability of counterfeit medicines, entering countries through porous national borders, and the lack of effective enforcement of legislation to ensure the supply of quality medicines further exacerbates the growing problem of AMR.

## IMPLEMENTATION OF NAPS IN RESPONSE TO AMR

After the declaration of AMR as a major global threat in 2015, the tripartite United Nations organizations (World Health Organisation [WHO], Food and Agriculture Organisation [FAO], and Office International des Epizooties [OIE]) adopted a common global action plan to combat AMR. The 194 member states of the WHO committed to integrating the 5 objectives and corresponding actions of the global action plan into national action plans (NAPs) to combat and prevent AMR. The 5 main objectives of the global action plan are to (1) improve awareness and understanding of AMR through effective communication, education, and training; (2) strengthen the knowledge and evidence base through surveillance and research; (3) reduce the incidence of infection through effective sanitation, hygiene, and infection prevention measures; (4) optimize the use of antimicrobial medicines in human and animal health; and (5) develop the economic case for sustainable investment in the fight against AMR. Most countries in SSA have only managed partial or phased implementation of their NAPs [47], and there is a need for increased awareness and action to address the problem of AMR in the region. By 2019, only 10 member states in the region had fully developed NAPs [48], and governments have still not committed adequate funds to implement the 5 key objectives to reverse the AMR trends we see currently.

This is a major challenge, as funding priorities usually change with onset of other emerging infections, such as the ongoing coronavirus disease 2019 pandemic. The implementation of all 5 key objectives of the NAPs are a crucial step toward addressing the problem of AMR. We require a strong government commitment and collaborative actions across the different sectors to establish a clear road map toward the gradual but compressive implementation of NAPs coupled with sustainable investment for action.

## CONCLUSIONS

The problem of AMR in SSA is increasingly becoming a threat to effective management and control of infectious diseases. To effectively lobby governments to invest in AMR prevention and control practices, it will be important to perform self-evaluations on the pertinent issues. Do we have representative data on AMR in healthcare-associated infections? These infections form an important reservoir for R genes, especially those carried on mobile genetic elements. Do we have adequate data on the economic cost of AMR that can persuade policy makers to act? Most countries have some data on AMR, but how about data on antimicrobial use (quantities and patterns), which is equally important? What are the key socioeconomic drivers of antimicrobial use? It will be crucial to enhance stewardship programs in healthcare facilities, as these will be important in mitigating practices that contribute to overuse and abuse of antimicrobials.

In addition, most countries in SSA have implemented infection prevention and control policies and guidelines as part of their NAPs, and this is playing a key role in reducing the burden of diseases and thus AMR, but these efforts must be scaled up to have a bigger impact. In a truly one-health spirit, we are yet to understand the role of environment as a contributor to AMR in communities, and efforts to include this ecosystem must be prioritized.

In addition, we have several WHO-approved vaccines (oral cholera vaccines [Dukoral, Shanchol, and Euvichol-Plus] and typhoid vaccines [Typhim Vi and Vivotif]) that could be deployed for short to medium term intervention for control and management of the key endemic enteric diseases in the region. Disease prevention is key to reducing burden of disease in endemic settings and directly tackles the problem of AMR. As SSA is facing an unprecedented rise in AMR against commonly available antimicrobials where alternative treatment options are either unavailable or too expensive for most of the population, we must promote the application of multiple measures to stem the rising tide in AMR and prolong the effectiveness of commonly available antimicrobials.

## Notes

***Acknowledgments.*** The authors thank the field and laboratory staff of the Mukuru kwa Njenga and Mukuru kwa Ruben clinics and the City Council Clinic, Embakasi Clinic and Centre for Microbiology, Kenya Medical Research Institute, for their contribution to data collection and laboratory analysis.

***Financial support.*** This work was supported by the National Institute of Allergy and Infectious Diseases, National Institutes of Health (grant R01AI099525) and the Wellcome Trust (grant 215675-Z-19-Z to S. K.).

***Potential conflict of interest.*** All authors: No reported conflicts. All authors have submitted the ICMJE Form for Disclosure of Potential Conflicts of Interest. Conflicts that the editors consider relevant to the content of the manuscript have been disclosed.

Previously presented: 15th Asian Conference on Diarrheal and Nutrition, Dhaka, Bangladesh, 28–30 January 2020.
